# TGF**β**1 Controls PPAR**γ** Expression, Transcriptional Potential, and Activity, in Part, through Smad3 Signaling in Murine Lung Fibroblasts

**DOI:** 10.1155/2012/375876

**Published:** 2012-09-10

**Authors:** Allan Ramirez, Erin N. Ballard, Jesse Roman

**Affiliations:** ^1^Division of Pulmonary, Critical Care, and Sleep Disorders, Department of Medicine, University of Louisville Health Sciences Center, Louisville, KY 40202, USA; ^2^Department of Pharmacology and Toxicology, University of Louisville Health Sciences Center, Louisville, KY 40202, USA; ^3^Pulmonary Section, Medicine Service, Robley Rex Veterans Affairs Medical Center, Louisville, KY 40202, USA

## Abstract

Transforming growth factor **β**1 (TGF**β**1) promotes fibrosis by, among other mechanisms, activating quiescent fibroblasts into myofibroblasts and increasing the expression of extracellular matrices. Recent work suggests that peroxisome proliferator-activated receptor **γ** (PPAR**γ**) is a negative regulator of TGF**β**1-induced fibrotic events. We, however, hypothesized that antifibrotic pathways mediated by PPAR**γ** are influenced by TGF**β**1, causing an imbalance towards fibrogenesis. Consistent with this, primary murine primary lung fibroblasts responded to TGF**β**1 with a sustained downregulation of PPAR**γ** transcripts. This effect was dampened in lung fibroblasts deficient in Smad3, a transcription factor that mediates many of the effects of TGF**β**1. Paradoxically, TGF**β**1 stimulated the activation of the PPAR**γ** gene promoter and induced the phosphorylation of PPAR**γ** in primary lung fibroblasts. The ability of TGF**β**1 to modulate the transcriptional activity of PPAR**γ** was then tested in NIH/3T3 fibroblasts containing a PPAR**γ**-responsive luciferase reporter. In these cells, stimulation of TGF**β**1 signals with a constitutively active TGF**β**1 receptor transgene blunted PPAR**γ**-dependent reporter expression induced by troglitazone, a PPAR**γ** activator. Overexpression of PPAR**γ** prevented TGF**β**1 repression of troglitazone-induced PPAR**γ**-dependent gene transcription, whereas coexpression of PPAR**γ** and Smad3 transgenes recapitulated the TGF**β**1 effects. We conclude that modulation of PPAR**γ** is controlled by TGF**β**1, in part through Smad3 signals, involving regulation of PPAR**γ** expression and transcriptional potential.

## 1. Introduction

Transforming growth factor *β*1 (TGF*β*1) is a pleomorphic growth factor with anti-inflammatory and profibrotic properties that has been implicated in many forms of natural and experimental tissue fibrosis [1]. In lung, TGF*β*1 is produced by epithelial cells, alveolar and tissue macrophages, and fibroblasts after exposure to injurious agents such as silica, bleomycin, hyperoxia, and paraquat among others [2]. A key role for TGF*β*1 in lung fibrosis has been confirmed in studies showing the development of lung fibrosis in animals transfected with TGF*β*1-producing adenovirus, and by work demonstrating inhibition of experimental lung fibrosis by interventions targeting TGF*β*1 or its downstream signals [3, 4].

The profibrotic effects of TGF*β*1 are mostly, but not entirely, mediated by intracellular signals triggered by the transcription factor Smad3 [5]. TGF*β*1/Smad3 signaling stimulates connective tissue expression and epithelial-mesenchymal transition, events considered key to the development of lung fibrosis [6]. In fibroblasts, TGF*β*1/Smad3 signaling stimulates their transdifferentiation into myofibroblasts and their expression of matrix genes like fibronectin and collagens [7]. Importantly, knockdown of the TGF*β*1/Smad3 signaling pathway inhibits experimental lung fibrosis [8]. In a model of immune-mediated airway fibrosis, we demonstrated inhibition of myofibroblast transdifferentiation and matrix deposition in animals deficient in Smad3 [9].

Considering its importance in the development of lung fibrosis, research directed at investigating the factors that control TGF*β*1/Smad3 signaling has intensified. This research has led to the exploration of *peroxisome proliferator-activated receptor *γ* (PPAR*γ*)*, a member of the ligand-activated nuclear hormone receptor superfamily of transcription factors that is known for its ability to regulate glucose and lipid metabolism and that has been implicated in insulin sensitivity, atherosclerosis, and inflammation [10, 11]. Preliminary studies in our laboratory suggested that PPAR*γ* inhibits the effects of TGF*β*1 through direct interactions with Smad3 (Ramirez et al., unpublished observations). Furthermore, others have demonstrated protection against TGF*β*1-induced myofibroblast transdifferentiation in cells treated with PPAR*γ* activators [12].

These observations suggest a promising role for PPAR*γ* activators in the treatment of fibrotic lung disorders. However, we wondered if TGF*β*1 influences PPAR*γ* expression and/or activation in tissues. To test this idea, we examined the effects of TGF*β*1 on PPAR*γ* in murine primary lung fibroblasts and found that TGF*β*1, in part through Smad3 signaling, differentially controls PPAR*γ* expression levels, transcription, and activation. These observations suggest that TGF*β*1/Smad3 signaling triggers profibrotic events, while concomitantly influencing the expression of PPAR*γ*.

## 2. Materials and Methods

### 2.1. Cell Culture

NIH/3T3 fibroblasts were purchased from ATCC. Primary murine lung from Smad3-deficient mice and wildtype C57BL/6 were generated and maintained as previously described [7, 9]. Primary lung fibroblasts were used between passages 2–10 in all experimental conditions. Where indicated, serum-starved fibroblasts were first pretreated for 1 hour in the presence or absence of TGF*β*1 (10 ng/mL) (R&D Systems, Minneapolis, MN, USA) followed by incubation with 10 *μ*M of troglitazone (Cayman Chemical, Ann Arbor, MI, USA) for the specified time. The studies were approved by the institutional animal research review committee.

### 2.2. Western Blots

Whole cell extracts were processed and analyzed as described [7, 9] with antibodies to phospho-PPAR*γ* (Millipore, Billerica, MA, USA) and PPAR*γ* protein (Cell Signaling Technology). *β*-Actin was used as control (Sigma, St. Louis, MO, USA).

### 2.3. Reverse Transcription-Polymerase Chain Reaction

Total RNA was isolated and tested as previously described [7]. Murine forward and reverse primers for PCR reactions were based on GenBank published sequences and are as follows: *PPAR*γ** (5′-GAC CAC TCG CAT TCC TTT-3′; 5′-CCA CAG ACT CGG CAC TCA-3′) and *28s rRNA* (5′-TTG AAA ATC CGG GGG AGA-3′; 5′-ACA TTG TTC CAA CAT GCC AG-3′). Amplicons were resolved on 1% agarose gels, stained with ethidium bromide, and visualized with a UV transilluminator.

### 2.4. Immunofluorescence Microscopy

A phospho-PPAR*γ* antibody was applied to paraformaldehyde-fixed, Triton X-100-permeablized cells at 4°C overnight followed by an Alexa Fluor 555-labeled anti-rabbit secondary antibody (Invitrogen). Slides were cover-slipped with ProLong Gold mounting medium (Invitrogen) and viewed under epifluorescence microscopy (Olympus BX41, Melville, MY, USA). Images were captured using MagnaFire 2.1 digital image acquisition software (Goleta, CA, USA).

### 2.5. Plasmids

Murine pCMX-PPAR*γ* [13] and the human PPAR*γ* promoter [14] were generous gifts from L. Jameson, (Northwestern University, Chicago, IL, USA) and C.M. Hart (Emory University, Atlanta, GA, USA), respectively. Flag-Smad3, AP2 (PPRE)-Luc, and T*β*RI (CA) were purchased from Addgene (numbers 14827, 8858, and 14833, resp., Cambridge, MA, USA).

### 2.6. Transfection and Reporter Studies

A calcium-phosphate transient transfection protocol was followed [15]. Briefly, at 50% confluence, cells were exposed to fresh growth media for 1 hour. DNA precipitate was applied for 24 hours. Cells were then washed, cultured for 6 hours in complete serum-free media, and treated as indicated. For reporter assays, treated cells were lysed using 5x Passive Lysis Buffer (Promega, Madison, WI, USA) and exposed to luciferase reagent, which was prepared according to Dyer et al. [16]. Luminescence was measured with a Thermo Luminoskan Ascent luminometer (Waltham, MA, USA) and normalized to Renilla activity [16].

### 2.7. Data Analysis

Western blotting and RT-PCR experiments were performed in duplicates and repeated at least three times to ensure consistency. Reporter and proliferation data were also repeated thrice each with 3-4 replicates per experiment. All results are presented as mean ± SE. GraphPad Prism v3.0 was used to analyze data by one-way ANOVA computation with Tukey's multiple comparisons test. A *P* value of 0.05 was considered significant.

## 3. Results

### 3.1. Effects of TGF*β*1 on PPAR*γ* Expression

**Figure 1:**
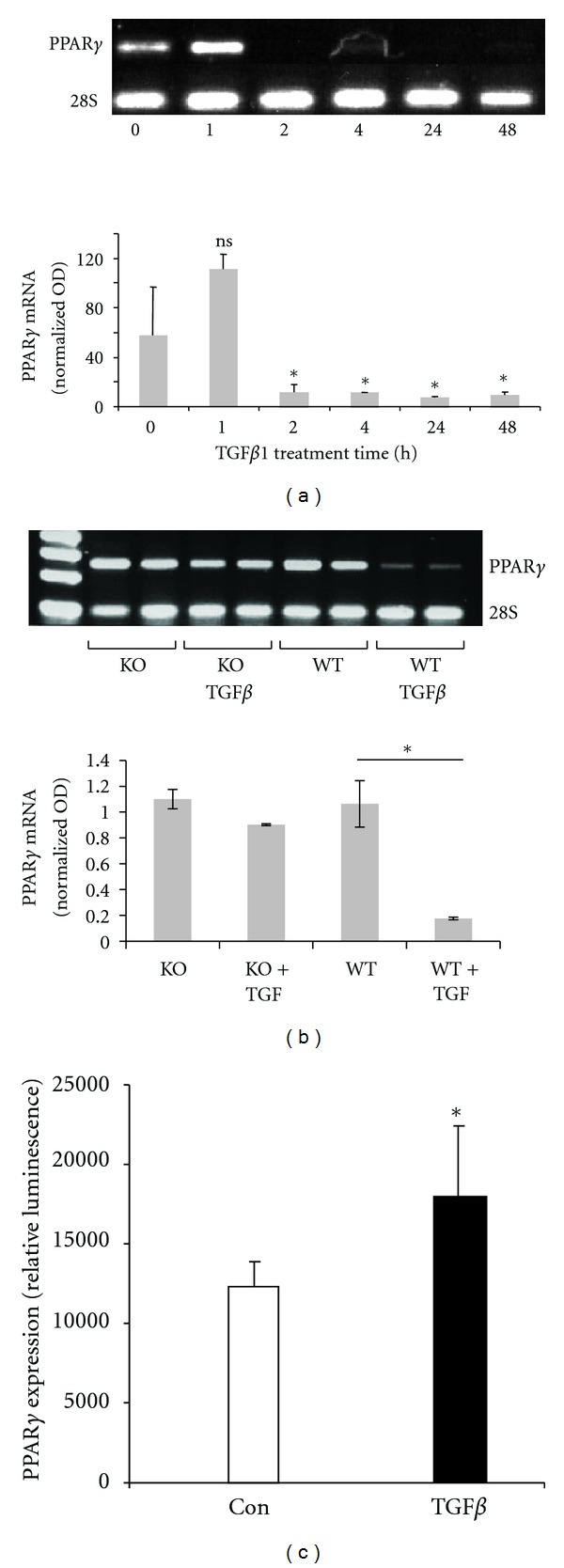
TGF*β*1 modulates PPAR*γ* expression, in part, via Smad3 signaling. (a) TGF*β*1 and PPAR*γ* mRNA expression. Murine primary lung fibroblasts were stimulated with TGF*β*1 (10 ng/mL) for the indicated times. The treated cells were then submitted for analysis by RT-PCR for PPAR*γ*. (b) Role of Smad3. Primary lung fibroblasts were isolated from the lungs of Smad3-deficient and wildtype mice and incubated with TGF*β*1 (10 ng/mL) for 24 hours. Transcripts of PPAR*γ* mRNA were detected using RT-PCR. *P < 0.05. (c) Regulation of PPAR*γ* gene transcription. NIH/3T3 fibroblasts were transiently transfected with a luciferase reporter driven by the full-length PPAR*γ* promoter and incubated with TGF*β*1 (10 ng/mL). Cellular extracts were analyzed after 24 hours for luminescence. Luciferase activity was normalized to Renilla with data shown as fold change ± SE relative to control. *P < 0.05.

 To begin to evaluate the effects of TGF*β*1 on PPAR*γ* expression, we first tested for PPAR*γ* gene transcription in lung fibroblasts. After a small and nonsignificant increase in PPAR*γ* expression, primary lung fibroblasts displayed a dramatic downregulation of PPAR*γ* mRNA expression beginning after an hour of exposure to TGF*β*1 and persisting for at least 48 hours (Figure 1(a)). To determine a mechanism by which TGF*β*1 might regulate PPAR*γ* expression, we examined the role of the transcription factor and TGF*β*1 intracellular transducer, Smad3. For this, primary lung fibroblasts were harvested from the lungs of Smad3-deficient mice and wildtype mice from the same genetic background and were cultured in the presence or absence of TGF*β*1 (Figure 1(b)). In wildtype primary lung fibroblasts, TGF*β*1 downregulated PPAR*γ* mRNA expression as previously demonstrated. However, this effect was greatly blunted in cells lacking Smad3, suggesting that Smad3 signaling is responsible for much of the inhibition of PPAR*γ* gene expression observed in TGF*β*1-treated fibroblasts.

To test if the inhibition of PPAR*γ* expression by TGF*β*1/Smad3 signaling occurred at the level of the gene promoter, NIH/3T3 fibroblasts were transfected with full-length human PPAR*γ* promoter ligated to a luciferase reporter (Figure 1(c)). However, instead of inhibiting PPAR*γ* expression, we observed that TGF*β*1 stimulated activity of the PPAR*γ* gene promoter.

### 3.2. Effect of TGF*β*1 on PPAR*γ* Phosphorylation

**Figure 2:**
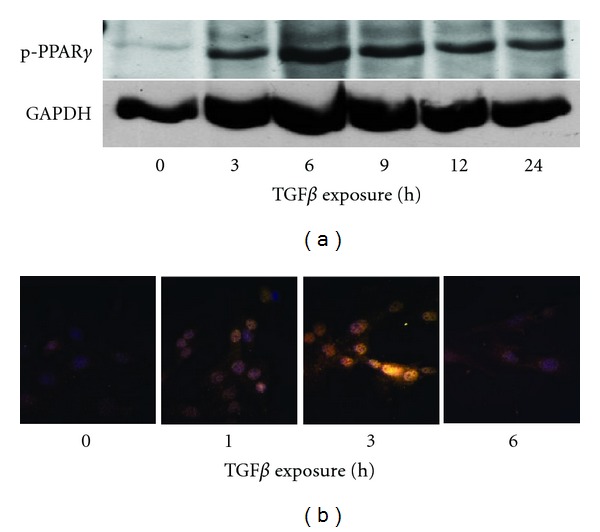
TGF*β*1 induces the phosphorylation of PPAR*γ*. Primary lung fibroblasts were treated with TGF*β*1 (10 ng/mL) for the indicated times. Phosphorylation of PPAR*γ* was detected by Western blot (a) and immunofluorescence microscopy (b) using a phospho-specific antibody.

Next, we determined if TGF*β*1 could induce PPAR*γ* posttranslational modifications, namely, phosphorylation, given TGF*β*1's known ability to activate intracellular signaling molecules. To this end, primary lung fibroblasts were treated with TGF*β*1 at several different time points and examined for phospho-PPAR*γ* with an antibody that specifically detects phosphorylation at serine 82 of PPAR*γ*1 (corresponding to serine 112 of PPAR*γ*2). By Western blot, phosphorylation of PPAR*γ* was detectable within 3 hours of TGF*β*1 exposure, with maximal effects at 6 hours, but persistent up to 24 hours after stimulation when the experiment ended (Figure 2(a)). In parallel experiments, phospho-PPAR*γ* was visualized by immunofluorescence in the nuclear compartment one hour after initiation of TGF*β*1 treatment, peaked three hours later, but was mostly absent by six hours (Figure 2(b)).

### 3.3. Effects of TGF*β*1 on PPAR*γ*-Dependent Gene Expression

**Figure 3:**
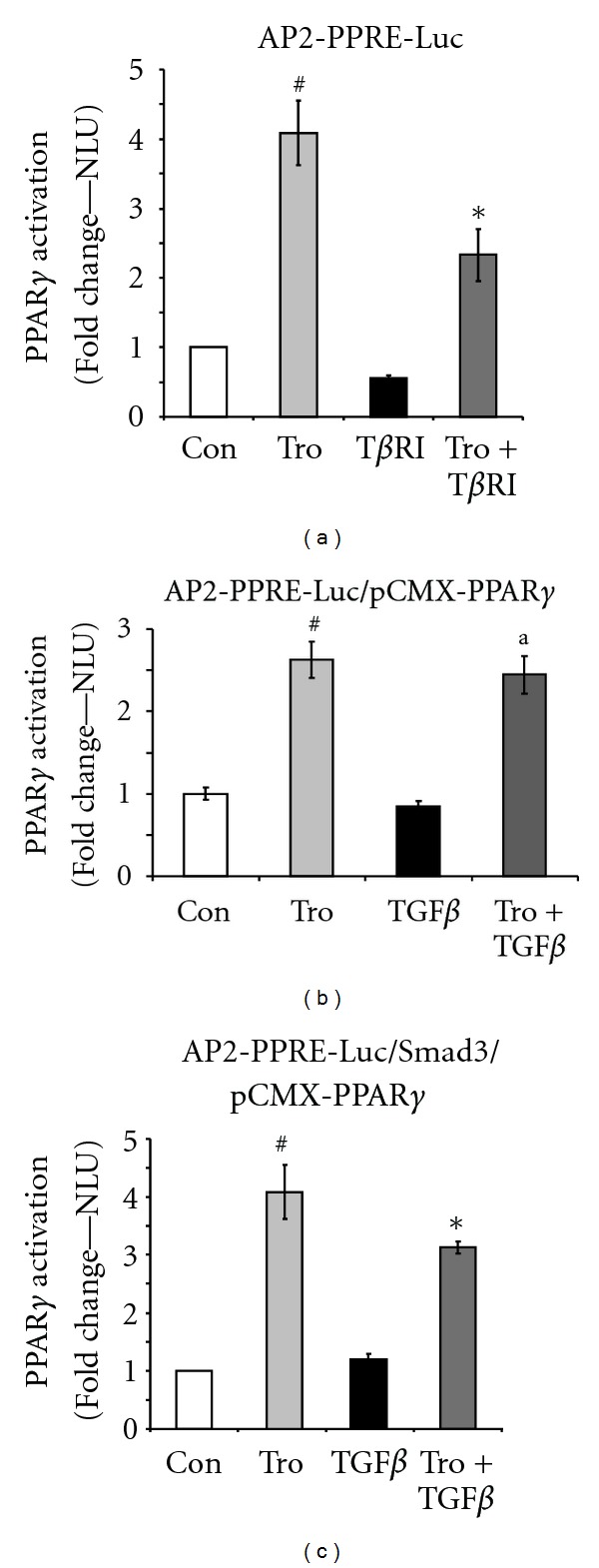
TGF*β*1 signaling inhibits the transcriptional activity of PPAR*γ*. PPAR*γ* transcriptional activity was determined with luciferase assays in NIH/3T3 fibroblasts bearing a PPAR*γ* response element-luciferase reporter (AP2-PPRE-Luc). Luciferase activity was normalized to Renilla with data shown as fold change ± SE relative to control. ^#^ *P* < 0.001 compared to control. *P < 0.001 compared to troglitazone. ^a^ *P*: ns compared to troglitazone. (a) Effects of troglitazone and TGF*β*RI. PPRE-containing NIH/3T3 fibroblasts were treated with troglitazone (Tro 10 *μ*M) and/or cotransfected with a constitutively active type I TGF*β* receptor (TGF*β*RI). At 24 hours, cells were analyzed for luciferase activity. (b) Overexpression of PPAR*γ* gene. The full-length PPAR*γ* gene and a PPRE were cotransfected into NIH/3T3 fibroblasts, which were then pretreated with TGF*β*1 (10 ng/mL) for 1 hour, followed by troglitazone (10 *μ*M) for 24 hours. (c) Role of Smad3. Smad3 and PPAR*γ* were coexpressed in NIH3T3 fibroblasts with a PPRE. Cells were exposed to TGF*β*1 (10 ng/mL) for 1 hour and then stimulated with troglitazone (10 *μ*M) for 24 hours.

 We then examined the functional effects of TGF*β*1 signaling on the transcriptional capability of PPAR*γ* after activation by the PPAR*γ* ligand, troglitazone. These experiments were conducted in NIH/3T3 fibroblasts containing a luciferase reporter driven by a PPAR*γ* response element. In other words, luciferase induction indicates stimulation of PPAR*γ*-dependent gene expression. As shown in Figure 3(a), troglitazone stimulated PPAR*γ* activation as demonstrated by the ability of troglitazone to induce PPAR*γ*-dependent gene transcription. This effect was blunted by the overexpression of a constitutively-active type I TGF*β* receptor, that is, T*β*RI or activin-like kinase 5 (ALK5), suggesting a role for TGF*β*1 receptor activation in the TGF*β*1-dependent repression of gene transcription by PPAR*γ*. However, the inhibition of transcriptional ability of PPAR*γ* by TGF*β*1 signaling was overcome by the forced overexpression of the full-length PPAR*γ* gene (Figure 3(b)). As expected, when a Smad3 transgene was cotransfected with the PPAR*γ* transgene in stoichiometrically equivalent amounts, TGF*β*1 was again able to partially offset activation of a PPAR*γ*-responsive gene promoter induced by troglitazone (Figure 3(c)). These data indicate that stimulation of TGF*β*1-dependent pathways, via activation of TGF*β*1 receptors and Smad3 signaling, regulates the activity of PPAR*γ* on gene transcription.

## 4. Discussion

 Our studies suggest that TGF*β*1 influences the expression and activity of PPAR*γ* with potential implications to lung inflammation and fibrosis. PPARs are recognized as versatile members of the ligand-activated nuclear hormone receptor superfamily of transcription factors that includes receptors for steroids, thyroid hormone, retinoic acid, and vitamin D among others [10, 11]. PPARs are considered to play key roles in diverse physiological processes ranging from lipid metabolism to inflammation and have been implicated in diseases such as cancer, atherosclerosis, and lung injury [17]. Three subtypes of PPARs have been identified and cloned: PPAR*α*, PPAR*β*/*δ*, and PPAR*γ*. Of the three PPARs identified to date, PPAR*γ* represents the most promising PPAR target in lung diseases in view of emerging reports implicating this molecule in various pulmonary processes [17]. Importantly, PPAR*γ* has been described as a negative regulator of macrophage function since its activation suppresses the production of inflammatory cytokines, chemokines, metalloproteinases, and nitric oxide [18]. These PPAR*γ*-mediated anti-inflammatory effects are not restricted to monocytic cells as treatment with PPAR*γ* agonists results in inhibition of cytokine and chemokine production in other cells [18–20]. More recently, it has been reported that PPAR*γ* activators inhibit TGF*β*1-induced myofibroblast transdifferentiation [12].

 In diseased tissues, PPAR*γ* expression has been shown to relate inversely with that of TGF*β*1 [21]. Thus, it appears that the balance between TGF*β*1 and PPAR*γ* may determine, among other factors, whether fibrogenesis predominates after tissue injury. However, in many patients and in experimental models, endogenous PPAR*γ* is unable to counter the effects of TGF*β*1. We reasoned that tissue injury results in the expression of factors capable of inhibiting PPAR*γ* expression or of blunting its antifibrotic effects. We further hypothesized that TGF*β*1 itself could directly influence PPAR*γ* expression. The observations reported here suggest that this is indeed the case. We observed that TGF*β*1 has an early, but transient, inductive effect on PPAR*γ* mRNA expression in primary lung fibroblasts; this effect was likely caused by the ability of TGF*β*1 to induce PPAR*γ* gene transcription (Figure 1). This early effect was associated with PPAR*γ* mRNA translation into protein and phosphorylation of the PPAR*γ* protein. However, this effect was later associated with profound inhibition of PPAR*γ* mRNA accumulation. The exact mechanisms responsible for this late effect are unknown, but increased mRNA degradation is likely. Based on these observations, we postulate that TGF*β*1 expression and/or activation in injured tissues is associated with early induction of a counterregulatory factor, PPAR*γ*. However, persistence of TGF*β*1 expression/activation results in late inhibition of PPAR*γ* mRNA accumulation. It should be highlighted that this “biphasic” effect of TGF*β*1 on PPAR*γ* (early stimulation and late repression) has been observed by others when studying the actions of TGF*β*1 on PPAR*γ* expression in vascular smooth muscle cells [22]. When studying these effects, one must consider cell type and other factors that could potentially affect the responses observed. For example, others have demonstrated that TGF*β* increases PPAR*γ* expression in H460 cells, but not in Ch27 cells, whereas nuclear accumulation of p-Smad3 was only observed in CH27 cells [23].

 Interestingly, despite late inhibition of PPAR*γ* mRNA accumulation, we observed persistent PPAR*γ* phosphorylation at least 24 hours after TGF*β* stimulation (Figure 2). This, however, did not correlate with persistent nuclear localization of PPAR*γ* as demonstrated by cytochemistry (Figure 2(b)). The mechanisms responsible for these events remain unclear.

 We also studied the role of Smad3 signaling in the effects observed. We found that Smad3 appears to mediate, at least in part, the effects of TGF*β*1 since cells deficient in Smad3 showed blunting of the TGF*β*1 downregulatory effect on PPAR*γ*. This was not surprising considering that Smad3 mediates many of the effects of TGF*β*1 in tissues. These data are consistent with observations made in dermal fibroblasts [21]. In other work, we reported that activation of P38 MAPK plays a role in TGF*β*1-induced myofibroblast transdifferentiation [7], and others have documented the interplay between TGF*β*1 and MAPKs in other systems [24]. Lin et al. (2011) reported that TGF*β*-induced expression of PPAR*γ* was related to activation of p38, but this was tested in H460 carcinoma cells [23]. Interestingly, they also showed that PPAR*γ*1 can bind Smad3 and p-Smad3. Zheng and Chen, on the other hand, reported that exogenous TGF*β* inhibits PPAR*γ* expression in activated hepatic stellate cells and this appeared mediated through Smads [25]. Furthermore, and consistent with our data, blocking TGF*β* signaling by dominant negative type II TGF*β* receptor increased PPAR*γ*. Choy and Derynck (2003) reported that TGF*β* inhibits adipogenesis by signaling through Smad3 which, in turn, interacts with C/EBPs leading to transcriptional inhibition of the PPAR*γ*2 promoter [26].

 The studies described above suggest that TGF*β*1 controls PPAR*γ* transcription, mRNA accumulation, and protein phosphorylation in differential ways. Considering the many points at which TGF*β*1 could influence PPAR*γ* expression and activation, these studies did not allow us to predict the overall effects of TGF*β*1 on PPAR*γ* function. This critical issue was addressed by testing cells transfected with a PPAR*γ*-responsive element that is transcribed only when activated PPAR*γ* enters the nucleus and interacts with target genes. To test the system, we first showed that troglitazone, a PPAR*γ* activator, stimulated PPAR*γ*-dependent gene expression (Figure 3(a)). Interestingly, expression of a constitutively active TGF*β*1 receptor, TGF*β*1RI, reduced the effect of troglitazone implicating this receptor in mediating the effects of TGF*β*1. Together, TGF*β*RII and TGF*β*RI form an activated ligand-receptor complex capable of stimulating downstream signals like Smads [27].

 As expected, the inhibition of transcriptional ability of PPAR*γ* by TGF*β*1 was overcome by the forced expression of the full-length PPAR*γ* gene. This is an important observation because it suggests that targeting the TGF*β*1/PPAR*γ* balance by enhancing PPAR*γ* activation may have potential therapeutic relevance. Several natural and synthetic compounds have been identified as activators of PPAR*γ*. The insulin-sensitizing antidiabetic drugs known as thiazolidinediones were the first compounds identified as PPAR*γ* agonists [28]; troglitazone, used in this report, is a thiazolidinedione, but it is no longer available commercially due to liver toxicity [29]. However, other thiazolidinediones are available and are being tested in clinical trials in different areas.

 Finally, we tested the role of Smad3 by cotransfecting cells with the Smad3 and PPAR*γ* transgenes in stoichiometrically equivalent amounts. As before, TGF*β*1 was able to partially offset activation of a PPAR*γ*-responsive gene promoter induced by troglitazone. These data indicate that stimulation of TGF*β*1-dependent pathways, via activation of TGF*β*1 receptors and Smad3 signaling, regulates the activity of PPAR*γ* on gene transcription.

 In conclusion, modulation of PPAR*γ* is intricately controlled by TGF*β*1, in part through Smad3 signals, involving tight regulation of PPAR*γ* expression levels and transcriptional potential. These mechanisms begin to explain how TGF*β*1 is able to overcome the anti-inflammatory and antifibrotic effects of PPAR*γ* and may have implications *in vivo*. The commercial availability of PPAR*γ* activators capable of tilting the TGF*β*1/PPAR*γ* balance to reduce, delay, and even reverse fibrosis raises the possibility of targeting PPAR*γ* in humans with fibrotic lung disease.
